# Dysfunctional mTORC1 Signaling: A Convergent Mechanism between Syndromic and Nonsyndromic Forms of Autism Spectrum Disorder?

**DOI:** 10.3390/ijms18030659

**Published:** 2017-03-18

**Authors:** Juliana Magdalon, Sandra M. Sánchez-Sánchez, Karina Griesi-Oliveira, Andréa L. Sertié

**Affiliations:** 1 Hospital Israelita Albert Einstein, Centro de Pesquisa Experimental, São Paulo 05652-900, Brazil; juliana.magdalon@einstein.br (J.M.); sandra.mabel@einstein.br (S.M.S.S.); karina.griesi@einstein.br (K.G.O.); 2 Departamento de Genética e Biologia Evolutiva, Instituto de Biociências, Universidade de São Paulo, São Paulo 05508-090, Brazil

**Keywords:** mTORC1 signaling pathway, ASD-related syndromes and nonsyndromic/idiopathic ASD, neuronal cell growth, axonal and dendritic morphogenesis, dendritic spine density and maturation, synaptic plasticity, mTORC1-targeted therapies

## Abstract

Whereas autism spectrum disorder (ASD) exhibits striking heterogeneity in genetics and clinical presentation, dysfunction of mechanistic target of rapamycin complex 1 (mTORC1) signaling pathway has been identified as a molecular feature common to several well-characterized syndromes with high prevalence of ASD. Additionally, recent findings have also implicated mTORC1 signaling abnormalities in a subset of nonsyndromic ASD, suggesting that defective mTORC1 pathway may be a potential converging mechanism in ASD pathology across different etiologies. However, the mechanistic evidence for a causal link between aberrant mTORC1 pathway activity and ASD neurobehavioral features varies depending on the ASD form involved. In this review, we first discuss six monogenic ASD-related syndromes, including both classical and potentially novel mTORopathies, highlighting their contribution to our understanding of the neurobiological mechanisms underlying ASD, and then we discuss existing evidence suggesting that aberrant mTORC1 signaling may also play a role in nonsyndromic ASD.

## 1. Introduction

Autism Spectrum Disorder (ASD) is among the most common developmental neuropsychiatric disorders and affects about 1 in 68 individuals [1]. It is characterized by impairments in two core domains: deficits in social communication and restricted, repetitive pattern of behavior or interests [2]. The presentation of symptoms is variable, ranging from mild to severe, and usually coexists with other psychiatric and medical conditions. There is a strong male bias in ASD, especially among individuals less severely affected (~4 males/1 female affected) [3,4].

ASD may be part of the clinical presentation of well-characterized genetic syndromes, hereinafter referred to as ASD-related syndromes, such as tuberous sclerosis complex (TSC) [5], fragile X syndrome (FXS) [6], Rett syndrome (RTT) [7,8], Angelman syndrome (AS) [9,10], phosphatase and tensin homolog (*PTEN)*-related syndromes [11], neurofibromatosis type 1 (NF1) [12], Timothy syndrome [13], 22q13.3 deletion syndrome [14], among others. These ASD-related syndromes, although representing only 5%–10% of all ASD cases, have contributed greatly to our understanding of ASD pathogenesis [15,16,17].

On the other hand, for most ASD cases, hereinafter called nonsyndromic ASD (NS-ASD)—even if additional phenotypic traits are present—to distinguish it from the well-defined ASD-related syndromes, the underlying causes remain unknown. Several twin and family studies have provided indisputable evidence for a genetic component underlying NS-ASD with heritability estimates ranging from 38% to 90% depending on the study parameters [18,19,20]. Recent high-throughput genomic techniques accompanied by large well-characterized cohorts of patients have identified a large number of rare and common variants for further characterization in relation to ASD [20,21,22,23], but several rare de novo and highly penetrant protein coding mutations appear to be sufficiently pathogenic to cause NS-ASD by themselves [24,25,26,27,28,29,30,31,32,33]. To date, a list of >200 ASD-risk genes categorized as “high confidence”, “strong candidate” and “suggestive evidence” can be found at the Simons Foundation Autism Research Initiative [34]. However, none of these genes accounts individually for more than 1%–2% of all cases of NS-ASD and, collectively, these forms of NS-ASD caused by highly penetrant mutations represent approximately 10%–20% of all cases, highlighting the enormous genetic heterogeneity of the disorder [35]. 

However, in spite of the high clinical and genetic heterogeneity of ASD, shared mechanisms between ASD-related syndromes and NS-ASD are being discovered and several mutated genes seem to converge on key biological pathways to give rise to ASD relevant symptoms [32,33,36,37,38]. One such pathway is the mechanistic target of rapamycin complex 1 (mTORC1) signaling cascade (Box 1, see below), which is a vital regulator of translation that impacts numerous cellular processes in the developing and mature brain [39,40]. Dysfunctional mTORC1 signaling has been described in several monogenic ASD-related syndromes, such as TSC, *PTEN*-associated ASD (PTEN-ASD), NF1, FXS [41,42], RTT [43] and AS [44,45]. Patient-derived cells and brain tissues as well as rodent models for these syndromes have been used to dissect the consequences of aberrant mTORC1 signaling in brain structure and function, as well as in particular aspects of cognition and behavior. In addition, recent findings suggest that *methyl-CpG binding protein 2* (*MECP2*) duplication syndrome [46] and *cyclin-dependent kinase-like 5* (*CDKL5*)-related syndrome [47] may also be associated with defective mTORC1 cascade activity; however, this needs further mechanistic exploration. Finally, more recently, dysregulation of mTORC1-dependent signaling, both upstream and downstream of its kinase activity, has also been observed in patients and animal models of NS-ASD of both known and unknown etiologies [42,48,49,50,51,52,53,54,55]. Nonetheless, our understanding of the mechanisms by which unbalanced mTORC1 signaling leads to NS-ASD is far less explored and many questions require further clarification, such as: (1) Is there enough evidence in the literature to support altered mTORC1 signaling in NS-ASD pathogenesis? (2) How large is the proportion of NS-ASD cases that show altered mTORC1 signaling? (3) Can the mechanistic insights gained from studying ASD-related syndromes be extrapolated to NS-ASD? (4) Will the putative mTORC1-targeted therapies that have been found to be effective for treating some ASD-related syndromes benefit at least a subgroup of NS-ASD patients?

Herein, we review the main mechanistic and therapeutic insights gained from studying six ASD-related syndromes with evidence for aberrant activation of mTORC1 pathway, and then discuss recent findings potentially linking mTORC1 signaling dysfunction to NS-ASD. A comparison of the main neuropathological features found in patients and rodent models of these ASD forms can be found in Table 1. Importantly, those phenotypes that were rescued by targeting mTORC1 pathway at different levels are also depicted in Table 1. All animal models described herein display ASD-relevant behaviors, except the *cytoplasmic FMR1 interacting protein 1* (*Cyfip1)* transgenic mice, in which ASD traits were not analyzed [52].

## 2. mTORC1 Signaling Pathway in Monogenic Autism Spectrum Disorder-Related Syndromes

### 2.1. Tuberous Sclerosis Complex (TSC)

**Figure 1:**
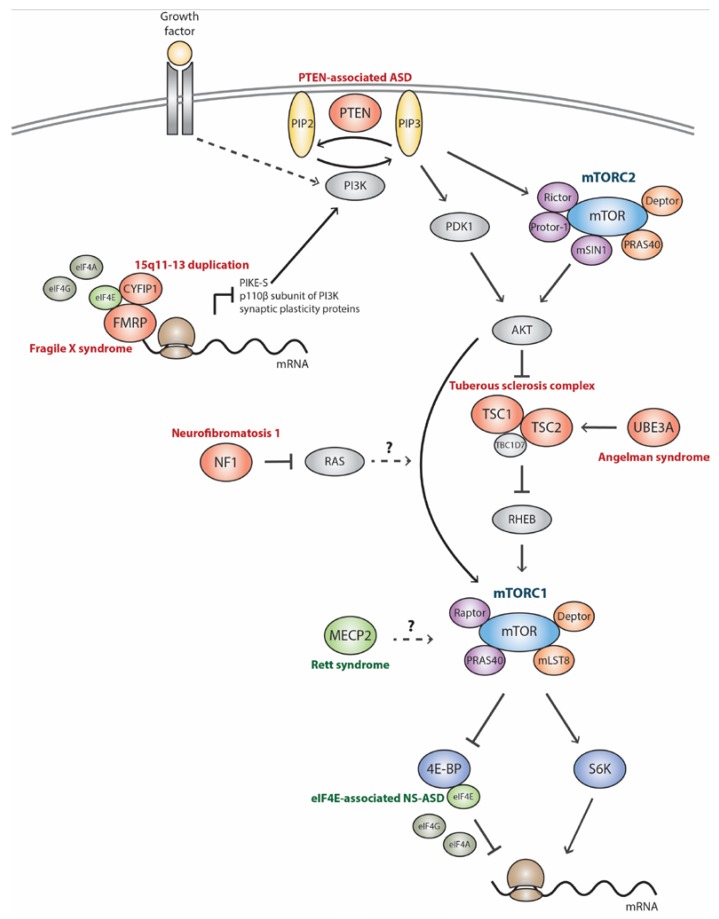
The mechanistic target of rapamycin complex 1 (mTORC1) signaling pathway. mTORC1 signaling components and proteins encoded by genes that inhibit (in red) or enhance (in green) mTORC1 pathway and cause autism spectrum disorder (ASD)-related syndromes and nonsyndromic ASD. Please see Box 1 for further details on mTORC1 signaling pathway. 4E-BP = eIF4E-binding protein; CYFIP1 = cytoplasmic FMR1 interacting protein 1; Deptor = DEP domain-containing mTOR-interacting protein; eIF = eukaryotic initiation factor; FMRP = fragile X mental retardation protein; MECP2 = methyl-CpG binding protein 2; mLST8 = mammalian lethal with SEC13 protein 8; mSIN1 = mammalian stress-activated protein kinase interacting protein 1; NF1 = neurofibromatosis 1; PDK1 = 3-phosphoinositide-dependent protein kinase 1; PI3K = phosphoinositide 3-kinase; PIKE = phosphoinositide 3-kinase enhancer; PIP = phosphatidylinositol; PRAS40 = proline-rich AKT substrate of 40 kDa; Protor-1 = protein observed with Rictor-1; PTEN = phosphatase and tensin homolog; Raptor = regulatory-associated protein of mTOR; RHEB = Ras homolog enriched in brain; Rictor = rapamycin-insensitive companion of mTOR; S6K = S6 kinase; TSC = tuberous sclerosis complex; UBE3A = ubiquitin-protein ligase E3A.

TSC (MIM#191100, #613254), a classical mTORopathy, is caused by loss-of-function mutations in the genes encoding TSC1 or TSC2 [56,57], which, together with TBC1D7 [58], form a complex that acts as a guanosine triphosphate (GTP)ase-activating protein for Ras homolog enriched in brain (RHEB) and negatively regulates mTORC1 [59,60] (Box 1, Figure 1). Therefore, constitutively active mTORC1 signaling constitutes the molecular basis of TSC [61,62]. The prevalence of ASD in TSC has been estimated to be ~36% [63]. Brain pathological features in patients include, in addition to epilepsy [64], benign proliferative lesions and focal malformation of cortical architecture termed cortical tubers, characterized by dysregulated mTORC1 activity, disruption of lamination, hyperexcitable synaptic network, giant cells, astrogliosis, reduced myelination, as well as dysplastic neurons with multiple and longer axons [65,66,67]. Several animal models with TSC downregulation, including constitutive heterozygous mutant mice and conditional knockout (KO) mice with *Tsc1/2*-deficiency in different cell types, have been used to shed light on the mechanisms by which TSC loss of function leads to brain alterations that, ultimately, converge on the neurocognitive impairments observed in TSC. Consistent brain functional and morphological abnormalities observed in these animals include seizures [68,69,70,71], larger brains [69,70,72], deficits in neuronal migration and cortical lamination [68,69,70,72,73,74], enlarged and dysplastic neurons [73,75,76,77] astrogliosis [68,69,70,73], reduced myelination [72,76], multiple and ectopic axons [78,79], enhanced excitatory network [65,80,81], and disrupted synaptic plasticity in the form of impaired hippocampal long-term potentiation (LTP) [82,83] and metabotropic glutamate receptor-mediated long-term depression (mGluR-LTD) [80,84] (Table 1). Notwithstanding, some conflicting results exist regarding normal/increased neurite length [78,79], reduced/normal/increased dendritic spine density and length [51,75,76,80,85,86] and increased spine head width/immature shape [75,85] (Table 1), possibly due to the use of different animal models or experimental conditions. Importantly, while it is still unknown whether increased neurite length and reduced LTP are dependent exclusively on mTORC1 overactivation, the majority of the other brain alterations in rodent models were partially or completely rescued or prevented by the mTORC1 inhibitor rapamycin [51,69,70,72,75,76,78,81,84,86] (Table 1), including astrogliosis, reduced myelination and ASD-relevant behaviors (not described in Table 1), even when treatment is begun in adulthood [77,87,88], demonstrating that they are dependent on mTORC1 overactivation. Notably, it was shown that cell-autonomous and non-cell autonomous mechanisms still not understood drive astrogliosis in *Tsc1*-deficient mice [68,69,70,73], and that neuron-specific deletion of *Tsc1* impairs oligodentrocyte maturation and myelination through a non-cell autonomously manner [89]. Interestingly, it was suggested that TSC2 deficiency affects neuronal migration through an abnormal crosstalk between mTORC1 and Reelin-Disabled 1 (Dab1) signaling pathways [74], and leads to increased spine density due to diminished postnatal mTORC1-mediated autophagy and spine pruning [51]. Paralleling these findings, it was shown that *Tsc1/2*-deficient neurons present mTORC1-dependent deficits in mitophagy, leading to mitochondria accumulation in cell soma and depletion from axon and pre-synaptic sites [79], which might also contribute to synaptic transmission dysfunction due to lack of adenosine triphosphate (ATP) [90,91]. On the other hand, an in vitro study has shown that *Tsc2*-deficient neurons have increased autophagy through 5' adenosine monophosphate (AMP)-activated protein kinase (AMPK) stimulation [92], which could potentially explain the controversial results regarding dendritic spine density observed in different studies. In addition, recent reports revealed mTORC1-dependent alterations in hippocampal protein synthesis in TSC mouse models, including decreased expression of synaptic proteins, such as the plasticity-related Arc protein required for mGluR-LTD [84], which could contribute to the impaired synaptic plasticity, but increased expression of stress-responsive proteins and anti-inflammatory cytokines [86], which may suggest an attempt to deal with the oxidative stress and inflammation that are frequently observed in the brain of individuals with ASD [93].

### 2.2. Phosphatase and Tensin Homolog-Associated ASD (PTEN-ASD)

Germline loss-of-function mutations in *PTEN* have been identified in patients with hamartoma tumor syndromes (PHTS) and in patients with ASD who also display macrocephaly with and without additional developmental features of PHTS (MIM#605309) [11,94,95]. Due to this reason and to the fact that higher lifetime risks for multiple cancers exist in patients with *PTEN* mutations, PTEN-ASD was included herein in the category of monogenic ASD-related syndromes. It has been estimated that the prevalence of *PTEN* mutations in patients with ASD and macrocephaly range from 7% to 27% [95,96,97]. *PTEN* encodes a lipid and protein phosphatase critical for modulating cellular growth, proliferation and survival [98]. PTEN counteracts the function of phosphoinositide 3-kinase (PI3K) and, similarly to TSC1/2, negatively regulates the mTORC1 pathway [99] (Figure 1). Therefore, *PTEN* deficiency is associated with constitutive activation of downstream AKT/mTORC1 pathways [41,100]. Except for macrocephaly, reports of brain pathological findings in patients are scarce and describe some structural abnormalities [101,102] and seizures in a few patients [103,104]. On the other hand, several heterozygous mice with constitutive *Pten* haploinsufficiency and conditional KO or knockdown mice with *Pten*-deficiency in different subsets of neuronal and glial cells have provided critical insights into the role of PTEN in the central nervous system (CNS), suggesting that it functions largely cell autonomously. Brain pathological features in these animals include seizures, macrocephaly, hypertrophy of both neurons and astrocytes throughout the brain [100,105,106,107,108,109,110]; enhanced glial cell number [105]; altered neuronal and glial migration [105,111,112]; severe abnormalities in myelination [107]; increased calibers, length and arborization of dendritic and axonal projections; increased dendritic spines density and length [100,106,107,108,109,110] (Table 1); and enhanced excitatory connectivity [108,109]. Importantly, treatment of *Pten*-deficient mice with rapamycin or pharmacological inhibition of S6 kinase 1 (S6K1) during early postnatal life prevented seizures, macrocephaly, aberrant neuronal migration, somatic, dendritic and axonal hypertrophy, increased neurite arborization and spine density (Table 1), enhanced excitatory connectivity, as well as reversed ASD-relevant symptoms [100,110,112], providing a causal link between elevated mTORC1 signaling and these neurobehavioral abnormalities in mouse models of *Pten* deficiency in the CNS (Table 1). Although alterations in glial cell growth, proliferation and migration, as well as in myelin production might be related to disease pathogenesis, further studies are needed to determine whether these abnormalities are linked to mTORC1 overactivation and affect ASD-relevant behaviors. Whereas increased/decreased number of immature-shaped spines [107,109] and reduced/increased LTP [107,113], decreased hippocampal mGluR-LTD [113] and cortical protein synthesis [110] are all neuropathological features also found in PTEN-ASD mouse models (Table 1), which might be in part linked to enhanced mTORC1 signaling activity as observed in TSC, a clear mechanistic link is still lacking and deserves deeper studies. 

### 2.3. Neurofibromatosis Type I (NF1)

NF1 (MIM#162200) is a tumor predisposition syndrome which may also exhibit cognitive impairments and ASD-like symptoms [114]. The prevalence of ASD in NF1 patients has been estimated to be ~18% [63]. NF1 is caused by loss-of-function mutations in the *NF1* gene, which encodes the RAS GTPase-activating protein termed neurofibromin. Consequently, *NF1* defect triggers RAS signaling activation [115,116], a key driver of cancer. The first studies suggesting mTORC1 involvement in NF1 showed that RAS can induce PI3K activation and subsequent TSC2 inhibition by AKT, increasing mTORC1 activity in *Nf1*-null mouse embryonic fibroblasts and astrocytes, as well as in cells derived from NF1 patient tumors [117,118]. Thereafter, however, it was shown that NF1 regulates glial cell proliferation and tumor growth in an AKT/mTORC1 dependent but TSC/RHEB independent manner [119] (Figure 1). Although only less than 10% of NF1 patients report seizures [120,121,122], several brain pathological features were frequently described in patients, such as macrocephaly [123,124] and reduced myelination [125,126], as well as in mouse models of the disorder, including larger brains [127], structural malformations [128,129,130], abnormal cerebellar neuronal migration [131,132], increased proliferation and protein synthesis in astrocytes [118,119,133], decreased neurite length [134,135], reduced dendritic spine density [136,137] and impaired LTP [138,139] (Table 1). Among those phenotypic alterations, it has been shown that rapamycin inhibited proliferation and protein synthesis in astrocytes [118,119] (Table 1), indicating that mTORC1 overactivation regulates astrocyte function in NF1 and is probably linked to glioma formation, such that pharmacological inhibition of mTORC1 suppresses tumor growth both in NF1 patients [140,141] and in mouse models [142,143]. Interestingly, not only *Nf1* loss of function in astrocytes may promote astrogliosis [118,119,133], but also neuron-specific *Nf1* deletion induces an increase in astrocyte number via a non-cell autonomous mechanism [144]. Nevertheless, further studies are required to unravel whether a defective glial proliferation affects social and other ASD-associated behaviors. Most of the other neuropathological features found in NF1 has not yet been associated with disrupted mTORC1 activity, and might also be due to mTORC1-independent functions of NF1. In fact, impaired cerebellar neuronal migration and LTP were shown to be dependent on extracellular signal-regulated kinase (ERK) signaling [131,132,139], whereas reduced neurite length is caused by defective cyclic AMP (cAMP) generation independently of RAS signaling [134,135].

### 2.4. Fragile X Syndrome (FXS)

FXS (MIM#300624) is considered the most commonly inherited cause of intellectual disability and a large percentage of individuals with FXS (~30%) are codiagnosed with ASD [63,145]. FXS is caused by transcriptional silence of the X-linked gene *FMR1* and loss of the protein product, fragile X mental retardation protein (FMRP) [146,147]. FMRP is an RNA-binding protein that negatively regulates the translation, stability and transport of several mRNAs, many of which encode proteins that are essential to synapse maturation, stabilization and elimination and that are well-studied ASD risk genes, such as *SH3 and multiple ankyrin repeat domains 3* (*SHANK3)*, *PTEN*, *TSC2*, *NF1*, *CYFIP1*, *Neuroligin 3* (*NLGN3*) and *Neurexin 1* (*NRXN1*) [147,148,149]. In addition to high incidence of epilepsy [150,151] and increased head circumference that may be present in FXS patients [152,153], consistent neuronal pathology includes increased dendritic spine density and overabundance of immature-shaped spines on neurons in various brain regions [154,155], which are thought to affect synaptic plasticity and network function (Table 1). In addition, it was shown that in vitro neurons derived from human FXS pluripotent stem cell lines show reduced cell size and neurite length [156,157,158] (Table 1). Accordingly, *Fmr1*KO mice, a model for human FXS, also exhibit a seizure phenotype [159,160], neurons with shorter neurite length [161] and with the atypical immature feature of FXS spines [160,162,163,164,165,166]. Additionally, it was shown that *Fmr1*KO mice exhibit altered neuronal migration and cortical circuitry [167], cerebellar astrogliosis [168], reduced cerebellar myelination [169], exaggerated hippocampal mGluR-LTD and protein synthesis [160,166,170,171,172], as well as overall brain hyperexcitability [173,174] (Table 1). Among these latter brain abnormalities, increased mGluR-LTD and protein synthesis were proved to play an important role in the neurological manifestations of FXS. In addition, less consistent results exist regarding decreased/normal neurite arborization in neurons derived from human FXS pluripotent stem cell lines [157,158], as well as normal/increased neuron size [175,176], increased/decreased/normal dendritic spine density [160,162,163,164,175,177] or length [165,175,178] and decreased/increased LTP [179,180,181] in *Fmr1*KO mice (Table 1). These discrepancies have been suggested to be due to differences in experimental conditions, brain regions examined, age and genetic background of the animals. It has been well documented that *Fmr1*KO mice exhibit upregulated mTORC1 signaling and elevated translation initiation complex formation in the brain [160,166,172], due at least in part to increased translation of the mRNAs encoding the p110β subunit of PI3K and its upstream activator PI3K enhancer (PIKE)-S, positive regulators of the mTORC1 pathway [172] (Figure 1). These findings suggest that in addition to its RNA-binding activity, FMRP also plays a role in the regulation of PI3K/mTORC1-mediated translation initiation. It is also noteworthy that although it has been suggested that mTORC1 signaling phosphorylates FMRP and inhibits its translation repressor activity [182], this finding was not confirmed in another study [183]. Importantly, pharmacological inhibition of either PI3K or mTORC1 rescues excessive synaptic protein synthesis in neurons from *Fmr1*KO mice [177]. In addition, genetic deletion of *S6K1* and pharmacological or genetic ablation of eukaryotic translation initiation factor (eIF) 4E phosphorylation, downstream targets of both ERK and mTORC1 pathways (Box 1; Figure 1), in *Fmr1*KO mice prevented dendritic spine morphology defects, synaptic plasticity alterations, exaggerated protein synthesis (Table 1) and ASD-associated behavioral phenotypes [160,166], providing a direct evidence that upregulated mTORC1 signaling and cap-dependent translation play a role in FXS pathophysiology.

### 2.5. Angelman Syndrome (AS)

Most cases of AS (MIM#105830) are caused by loss of function of the maternally-inherited *ubiquitin protein ligase E3A* (*UBE3A)* allele in neuronal cells [184,185], which encodes a protein that targets other proteins for degradation. This gene is localized on a cluster of imprinted genes on chromosomal region 15q11-13 such that *UBE3A* is paternally imprinted and silenced by a non-coding antisense transcript [186]. A substantial portion of AS patients meets criteria for ASD [187], and the prevalence of ASD in AS has been estimated to be ~34% [63]. Brain pathological features already described in AS patients include epilepsy [188,189], microcephaly [190] and reduced myelination [191,192]. These abnormalities have been also observed in mice with a maternal null mutation in *Ube3a* (AS mice) [193,194,195], which additionally exhibit abnormal spine morphology, reduced dendritic spine density and length [44,45,196], suggesting that deficient synaptic development may underlie the neurological aspects of AS. Moreover, an overall excitatory network, due to a more severe decrease in inhibitory than excitatory inputs [197], possibly contributes to the increased seizure susceptibility observed in AS patients. In vitro studies using *Ube3a* knockdown or *Ube3a*KO neurons from mice have also shown that *Ube3a* loss of function decreased dendrite arborization, disrupted dendrite polarity and reduced apical dendrite length [198,199]. In addition, disrupted synaptic plasticity in the form of impaired LTP in different brain areas [45,193,200] and enhanced hippocampal mGluR-LTD [201] were also observed in AS mice (Table 1). This is believed to be mainly caused by increased levels of Arc due to its reduced ubiquitination by the UBE3A proteins and, consequently, reduced degradation [202]. Importantly, recent studies have also observed that the increased levels of Arc may also be the result of increased mTORC1 signaling and its downstream target S6K1 activation in the cerebellum and hippocampus of AS mice, triggered by increased inhibitory phosphorylation of TSC2 in the absence of UBE3A [44,45], although the precise mechanism is unknown. In addition to increasing Arc levels and improving LTP deficits, rapamycin or an S6K1 inhibitor also ameliorated dendritic spine density and morphology in Purkinje and pyramidal cells (Table 1), and consequently, motor dysfunction and learning deficits in AS mice [44,45], suggesting that mTORC1 activity may also be affecting synaptic plasticity and function in AS patients. Given the fact that the association between mTORC1 and UBE3A deficiency has been only recently demonstrated, further studies are necessary to test whether the other brain abnormalities found in AS are dependent on mTORC1 overactivation and would benefit from mTORC1-targeted therapies.

### 2.6. Rett Syndrome (RTT)

RTT (MIM#312750) is a severe progressive neurodevelopmental disorder that manifests mostly in girls during early childhood after a typical perinatal development. Although RTT is no longer considered an ASD in Diagnostic and Statistical Manual of Mental Disorders, fifth edition (DMS-5) [2], children afflicted with RTT often exhibit ASD-like behaviors, and the prevalence of ASD symptoms in RTT has been estimated to be ~61% in female patients [63]. RTT is mainly caused by loss-of-function mutations in the X-linked gene *MECP2* [203,204], which encodes a methyl-CpG binding protein that controls gene expression and chromatin remodeling [205]. In addition to epilepsy [206,207] and reduced brain size [208,209], brain pathology in human patients that are thought to contribute to the neurocognitive deficits in RTT includes reduced neuronal size but increased neuronal cell density in several brain regions [210,211], evidence of cortical astrogliosis [212], decreased dendritic arborization and length [213,214], as well as decreased spine density and maturation in the cortex and hippocampus [215,216,217] (Table 1). These neuronal abnormalities have been consistently reproduced by several studies using genetically distinct rodent models of RTT [218,219,220,221,222,223,224,225], which additionally exhibit abnormal activity-dependent synaptic plasticity in the form of attenuated LTP and LTD [226,227], and reduced number of excitatory synapses in hippocampal neurons [228] (Table 1). Notably, studies in RTT mouse models have suggested that cell autonomous and non-cell autonomous mechanisms drive neuronal morphology and function [229,230,231]. In addition, recent in vitro models of RTT using *MECP2*-deficient neurons derived from human pluripotent stem cells have recapitulated many neurological features of RTT [232,233,234,235,236], and have also shown neuronal migration defects [236] (Table 1). Interestingly, in contrast to the majority of the ASD-associated mTORopathies, neurons from *Mecp2*^−/−^ and *Mecp2*^+/−^ mice [43], as well as from *MECP2*-deficient human pluripotent stem cells [233], show decreased mTORC1 signaling activity, transcription and protein synthesis rate. These findings suggest that mTORC1 signaling deviations in either direction can adversely affect neuronal connectivity, cognition and social behavior. Although the mechanism by which MECP2 enhances mTORC1 signaling is still unknown (Figure 1), the levels of brain-derived neurotrophic factor (BDNF) are lower in RTT mouse models, possibly resulting in lower activation of PI3K/mTORC1 pathways [43]. Treatment of neurons derived from *MECP2*-deficient human pluripotent stem cells with exogenous growth factors (insulin-like growth factor 1 [IGF-1] or BNDF) or genetically ablation of *PTEN*, promoted protein synthesis via enhancing PI3K/mTORC1 signaling activity and rescued the soma size and neurite complexity deficits [233] (Table 1). These findings suggest that defects in the global control of transcription and PI3K/mTORC1-mediated translation might be the underlying pathomechanisms by which MECP2 dysfunction leads to RTT, although additional mechanistic exploration are needed.

## 3. mTORC1 Signaling Pathway in Nonsyndromic/Idiopathic Autism Spectrum Disorder 

### 3.1. 15q11-13 Duplication (Dup15q)

Maternally inherited duplications at 15q11-13 (#MIN608636) is one of the most frequent and penetrant copy number variation in ASD, found in ~1%–2% of patients [237,238], suggesting that one or several genes from this region, when duplicated, can lead to ASD. Deletions at this same region give rise to Prader–Willi syndrome (PWS) or AS depending on whether the deletions are paternally or maternally inherited, respectively [239] and, as discussed above, ASD symptoms are usually reported in AS patients. Instability of this region is mediated by the presence of five low copy repeats, termed breakpoint (BP)1 through BP5. The PWS/AS critical region lies on the imprinted region between BP2 and BP3, and evidence from mouse models suggests that increased dosage of the *Ube3a* gene located in this region impairs excitatory synapse transmission and might underlie ASD-relevant behaviors [240]. It is noteworthy that, although duplications of paternal origin show low penetrance, a mouse model with a paternally inherited duplication of the BP2–BP3 interval (patDp/+ mice) displays behaviors associated with ASD [241], increased spine turnover [242] and impairment in cerebellar LTP [243] (Table 1). In addition, there is evidence suggesting that the more proximal non-imprinted region between BP1 and BP2 (15q11.2) is also a hot spot for ASD and that genes located in this region impact neurological and behavioral functions [244,245,246]. Among the four genes located between BP1 and BP2, *CYFIP1* became a prime candidate for a causal role in ASD [52,245]: it directly interacts with FMRP and with eIF4E and mediates the translational repression activity of FMRP in the brain [247], and also regulates actin polymerization and cytoskeleton remodeling through its interaction with the small GTPase Rac1 [248,249]. It was shown that CYFIP1 levels are increased in lymphoblastoid cells [52,250] and *postmortem* brain tissues (temporal cortex) [52] from ASD subjects with Dup15q, and its overexpression in cultured human and mouse neuronal cells leads to increased neuronal cell size, neurite arborization, as well as increased/decreased neurite length [52,251]. Similar abnormalities in neuronal cell size, neurite outgrowth and branching were also observed in transgenic mice overexpressing *Cyfip1*, which additionally exhibit increased spine density and number of mature spines in cortical neurons [52] (Table 1), defects previously shown to contribute to synaptopathology that drives ASD-related symptoms. However, further research should evaluate whether *Cyfip1* transgenic mice exhibits autistic traits. Importantly, evidence for increased mTORC1 signaling was observed in *postmortem* brains of ASD-Dup15q carriers (*n* = 3), as well as in embryonic *Cyfip1* transgenic mice and cultured mouse neuronal cells overexpressing *Cyfip1*. Pharmacological treatment of these cultured cells with rapamycin rescued the observed abnormalities in cell size, neurite length and branching [52]. Finally, evidence for hyperfunctional mTORC1 signaling was also observed in cultured stem cells from human exfoliated deciduous teeth (SHED) derived from one ASD patient with Dup15q [53]. Taken together these findings suggest that CYFIP1-mediated mTORC1 signaling overactivation may contribute to disease pathogenesis in ASD-Dup15 patients.

### 3.2. eIF4E-Associated NS-ASD (eIF4E-NS-ASD)

mTORC1 signaling phosphorylates eIF4E-binding proteins (4E-BPs) and releases them from eIF4E, allowing eIF4E to interact with eIF4G and eIF4A to form the eIF4F complex, a critical step in cap-dependent translation (Box 1; Figure 1). Interestingly, rare mutations in the promoter region of the *eIF4E* gene, which were suspected to enhance promoter activity, were found in few unrelated NS-ASD patients [48]. Later on, it was shown that transgenic mice overexpressing *eif4e* and mice lacking *4e-bp2* display increased dendritic spine density, altered synaptic plasticity, including augmented excitation, enhanced late-phase LTP and mGluR-LTD in the prefrontal cortex and hippocampus, as well as exaggerated brain cap-dependent translation [49,50] (Table 1). Treatment of *eif4e*-transgenic and *4e-bp2*KO mice with 4EGI-1, an inhibitor of eIF4E-eIF4G interaction, rescued protein synthesis and synaptic plasticity abnormalities (Table 1), as well as ASD-relevant behaviors [49,50]. These findings provided a causal link between NS-ASD and excessive cap-dependent translation, and suggest that this is one of the targets by which mTORC1 inhibitors reverse synaptic plasticity deficits and ASD symptoms. 

### 3.3. Idiopathic Autism Spectrum Disorder

Paralleling neuropathological observations from ASD-related syndromes, children with ASD of unknown etiology also frequently display increased risk for developing epilepsy [252,253], macrocephaly in early childhood [254,255], increased neuronal density in several brain regions but reduced number of Purkinje cells [255,256], normal/decreased neuronal size [255,257], altered neuronal migration [258,259], astrogliosis and microglial activation [260], altered myelination [261,262], increased dendritic spine densities on cortical neurons [51,263] and impaired LTP [264]. In addition, increased number of inhibitory synaptic connections was described in cultured neurons derived from patient-induced pluripotent stem cells (iPSCs) [265], and increased spine turnover was found in the BTBR inbred mouse strain that displays the core behavioral deficits of ASD [242]. Although these abnormalities are most probably caused by different etiological origins, recent studies have shown that there is a subgroup of idiopathic ASD with defects in mTORC1 signaling activity. Hyperactivation of mTORC1 pathway was observed in *postmortem* brains from adolescent patients with idiopathic ASD (*n* = 5) and was shown to impair autophagy and spine pruning during childhood and adolescence, leading to increased basal dendritic spine density and, therefore, enhanced excitatory connectivity [51] (Table 1). As discussed above, similar findings were also observed in TSC mouse models in the same study, suggesting that downregulation of mTORC1 signaling is required for postnatal spine pruning [51]. In addition, mTORC1 pathway upregulation was also observed in non-neuronal cells derived from idiopathic ASD patients, such as in cultured stem cells from SHED derived from 2 out of 12 patients [53], and in lymphoblastoid cell lines (LCLs) derived from 4 out of 58 patients (7% of the patient sample) [55]. Interestingly, in LCLs from one of these patients with elevated mTORC1 pathway activity, it was also observed increased expression of the p110δ subunit of PI3K and enhanced protein synthesis rates, which were corrected by a p110δ-specific inhibitor [55] (Table 1). Curiously, contrary to these findings, a study reported evidence for decreased mTORC1 signaling in *postmortem* brains from patients with idiopathic ASD (*n* = 11; aged 5–56 years, mean 20.1 years) [54] (Table 1). The high etiological heterogeneity in this group of patients may account for these observed discrepancies in the direction of mTORC1 signaling activation, and additional investigations are needed to provide further mechanistic understanding of the causal link between mTORC1 pathway dysfunction and ASD in this group.

## 4. Discussion

Research on both ASD-related syndromes and NS-ASD and their corresponding mouse models has shown that abnormalities in brain size and structure, neuronal size, migration and myelination, astrocyte proliferation, neurite and dendritic spine morphology, synapse plasticity, imbalanced synaptic excitation/inhibition, as well as dysregulated brain protein synthesis are all common features of ASD across different etiologies. Although aberrant mTORC1 pathway activation has been suggested as a convergent molecular mechanism in ASD etiopathology, the evidence supporting a causal relationship between abnormal mTORC1 signaling and these brain anatomical and physiological deficits, as well as behavioral alterations found in patients and/or animal models, varies greatly depending on the ASD form involved. Herein, we will discuss only the neurobehavioral abnormalities that were experimentally linked to mTORC1, which include those phenotypes that were rescued or prevented by modulating mTORC1 cascade activity at different levels.

Seizures, enlarged brain and neuron size, neuronal migration abnormalities and increased neurite arborization were mechanistically linked to enhanced mTORC1 signaling in mouse models of ASD-related syndromes caused by mutations in upstream negative regulators of the pathway, such as TSC [69,70,72,75,76,78] and PTEN-ASD [100,112]. Interestingly, overactivation of mTORC1 was also associated with increased neuronal size and neurite arborization in cultured neuronal cells overexpressing *Cyfip1*, located in the 15q11.2 ASD risk locus [52]. On the other hand, diminished mTORC1 pathway seems to be associated with decreased neuronal size and neurite arborization in RTT models [233]. These results suggest that an optimal level of mTORC1 signaling activation is required to maintain proper brain and neuron size, as well as neurite branching patterns, such that abnormalities in these morphological features of neurons may contribute to ASD pathogenesis.

Atypical number and/or length and/or morphology of dendritic spines were mechanistically linked to disinhibited mTORC1 signaling in different mouse models of ASD-related syndromes, including TSC [75,76,86], PTEN-ASD [100], FXS [160,166] and AS [44,45], as well as in NS-ASD models, such as *Cyfip1* transgenic mice [52], eIF4E-NS-ASD mice [49,50] and idiopathic ASD patients [51]. Importantly, a recent study reported that disruption of mTORC1-dependent macroautophagy reduces spine pruning and consequently increases spine density in neurons of individuals with TSC or idiopathic ASD [51]. It would be important to address whether defects in mTORC1-mediated autophagy also play a role in impaired developmental pruning of neuronal connections in other ASD models. Together these findings suggest that mTORC1-mediated abnormalities in dendritic spine number and structure are central in ASD pathogenesis across multiple underlying causes. 

Abnormal mTORC1-mediated brain protein synthesis was shown to play a role in the synaptic pathophysiology of TSC [84,86], FXS [160,166,177] and eIF4E-NS-ASD [49,50] mouse models. In addition, different responses for overactivated mTORC1 signaling in synaptic plasticity was observed depending on the ASD mouse model, i.e., mTORC1 may reduce mGluR-LTD in TSC [84] but enhance it in FXS [160,166] and eIF4E-NS-ASD [49,50], as well as decrease LTP in AS [44,45] and increase it in eIF4E-NS-ASD [49,50]. Interestingly, there is a tendency for those ASD forms with heightened mTORC1-dependent translation of synaptic proteins, such as FXS and eIF4E-NS-ASD, to display enhanced mGluR-LTD, whereas TSC, which show decreased mTORC1-dependent translation of synaptic proteins, exhibit impaired mGluR-LTD, suggesting that altered (either enhanced or reduced) mTORC1-mediated protein abundance of synaptic proteins, such as Arc, may influence mGluR-LTD and may be implicated in the synaptic defects and cognitive impairments associated with ASD pathogenesis across different genetic causes. Although PI3K/mTORC1-associated protein synthesis defects have also been observed in *Nf1*-deficient astrocytes [118], in *MECP2*-deficient human pluripotent stem cells (model of RTT) [233] and in lymphoblastoid cell lines from an idiopathic ASD patient [55], further investigation is required in order to unravel a potential link with synaptic plasticity abnormalities and ASD-like symptoms in these different ASD models. 

Finally, and perhaps most importantly, a direct role for overactivated mTORC1 signaling in ASD core symptoms is supported by multiple evidence in mouse models of TSC [77,87,88], PTEN-ASD [100], FXS [160,166] and eIF4E-NS-ASD [49,50], showing that pharmacological or genetic inhibition of mTORC1 cascade both upstream and downstream of its kinase activity rescues or attenuates ASD-relevant behaviors, highlighting the potential therapeutic value of drugs targeting this pathway for patients. Indeed, based on evidence for the benefit of rapamycin and similar drugs on neurobehavioral deficits in TSC mouse models, clinical trials aimed to evaluate the effect of mTORC1 inhibitors on neurocognition in TSC patients are currently underway [266]. 

Therapeutic approaches based on mTORC1 inhibition might benefit NS-ASD patients as well. There are evidences that individuals with Dup15q, which accounts for 1%–2% of all ASD cases, display aberrant mTORC1 pathway activation [52] and, thus, it would be interesting to develop preclinical and clinical studies to evaluate the ability of drugs acting on mTORC1 cascade to ameliorate neurobehavioral function in Dup15q. Lastly, some studies have described mTORC1 signaling defects in a sub-cohort of patients with idiopathic ASD [51,53,54,55], although further functional studies are required to provide additional and mechanistic proof of a causal link between mTORC1 abnormalities and ASD development in this group of patients. While it is currently difficult to estimate the proportion of patients with ASD of unknown etiology that show aberrant mTORC1 pathway, pilot studies using patient-derived non-neuronal cells [53,55] have opened up the exciting possibility of large-scale screens for mTORC1 signaling defects using more easily accessible patient biological material, which might be used to select those patients who could possibly benefit from treatments targeting mTORC1 pathway. It is noteworthy that mTORC1 signaling abnormalities may be caused by a variety of factors in this group, including genetic, epigenetic and environmental risk factors, which may further complicate clinical studies; however, in spite of these challenges, identifying a subgroup of patients that will benefit from mTORC1-targeted therapies will be of paramount importance.

## 5. Conclusions and Future Directions

A causal relationship has been established between disturbed activation of mTORC1 signaling pathway and several neurological abnormalities observed in different well-characterized monogenic syndromes with high prevalence of ASD (TSC, PTEN-ASD, FXS, AS and RTT), and preclinical studies have shown that modulation of mTORC1 signaling may provide promising avenues for the treatment of ASD-relevant symptoms. The emerging evidence for aberrant mTORC1 signaling activation in a subgroup of patients with nonsyndromic/idiopathic ASD also provides an exciting possibility for the treatment of behavioral and cognitive deficits in these patients as well. The findings that defective mTORC1 activity can also be detected in non-neuronal more easily accessible cells suggest that mTORC1 cascade components may potentially be used as biomarkers to identify those patients most likely to benefit from mTORC1-targeted therapies. However, given the etiological complexity of ASD in this group, additional studies are required to further explore the mechanistic relevance of mTORC1 pathway alterations to the disease.

Box 1 **mTOR signaling biology.**

mTOR is a large (predicted molecular weight 280 kD) serine/threonine kinase that can combine with protein binding partners to form one of two functionally distinct mTOR complexes: mTORC1 and mTORC2. In addition to mTOR, mTORC1 consists of regulatory-associated protein of mTOR (Raptor) and mammalian lethal with SEC13 protein 8 (mLST8), which are essential for mTORC1 function, as well as proline-rich AKT substrate of 40 kDa (PRAS40) and DEP domain-containing mTOR-interacting protein (Deptor), inhibitors of mTORC1 activity (Figure 1). Although most of mTORC1 actions are sensitive to rapamycin, mTORC2 is mostly insensitive to rapamycin and contains the core components mTOR, mLST8, Deptor, mammalian stress-activated protein kinase interacting protein 1 (mSIN1), rapamycin-insensitive companion of mTOR (Rictor) and protein observed with Rictor-1 (Protor-1) (Figure 1). Although much less is known about mTORC2 than is known for mTORC1, a growing amount of literature demonstrates a role for mTORC2 in cytoskeletal integrity and neuronal morphology [267,268]. To date, the majority of neurological disorders associated with mTOR signaling have been linked to mTORC1 [39,269]. In the presence of growth factors, such as insulin, the PI3K is activated and stimulates phosphatidylinositol (3,4,5)-trisphosphate (PIP3) production. PIP3 accumulation in the plasma membrane promotes AKT recruitment, phosphorylation and activation by 3-phosphoinositide-dependent protein kinase 1 (PDK1) and mTORC2. When active, AKT phosphorylates and inhibits TSC2 that, together with TSC1 and TBC1D7 [58], is part of the tuberous sclerosis complex (TSC) [270]. TSC functions as a GTPase-activating protein (GAP) toward RAS homolog enriched in brain (RHEB), stimulating the conversion of RHEB-GTP to RHEB-GDP and inactivating this protein. Therefore, the inhibition of TSC by AKT promotes RHEB activation, which then activates mTORC1 in the presence of amino acids [271,272]. Among several processes, mTORC1 inhibits autophagy and stimulates mRNA translation, which is dependent on the phosphorylation and activation of S6 kinase (S6K) and inhibition of eukaryotic translation initiation factor 4E (eIF4E)-binding proteins (4E-BPs), releasing it from eIF4E and enabling interaction with eIF4G and eIF4A to form the eIF4F translation initiation complex, a critical step in cap-dependent translation (Figure 1). In addition to insulin and amino acids, other signals modulate mTORC1 activity, such as levels of ATP, glucose and oxygen. Therefore, mTORC1 is considered a sensor of internal and external cues that maintains cellular homeostasis through modulation of anabolic and catabolic processes [273].

## Figures and Tables

**Table 1:** Main brain functional and morphological features found in patients and/or in vitro human pluripotent stem cells models and/or rodent in vivo and in vitro models of autism spectrum disorder (ASD)-related syndromes and nonsyndromic ASD forms with evidence for aberrant mechanistic target of rapamycin complex 1 (mTORC1) signaling pathway (please see the manuscript text for complete details and references). The phenotypes that were rescued and/or prevented by targeting mTORC1 pathway at different levels are indicated.

Phenotypes	ASD-Related Syndromes						Nonsyndromic or Idiopathic ASD		
	TSC	PTEN	NF1	FXS	AS	RTT	Dup15q	eIF4E-ASD	Idiopathic
**mTORC1 signaling**	↑	↑	↑	↑	↑	↓	↑	↑	↑/↓
**Seizures**	Present *^,1^	Present *^,1^	Present	Present	Present	Present	Present	ND	Present
**Brain size**	↑ *^,1^	↑ *^,1^	↑	↑	↓	↓	↓	ND	↑
**Neuron size**	↑ *^,1^	↑ *^,1,3^	ND	↑/↓/normal	ND	↓ *^,5,6^	↑ *^,1^ (*Cyfip1*)	ND	↓/normal
**Neuronal migration**	Abnormal *^,1^	Abnormal *^,1^	Abnormal	Abnormal	ND	Abnormal	Abnormal	ND	Abnormal
**Neurite arborization**	↑ *^,1^	↑ *^,3^	ND	↓/normal	↓	↓ *^,5,6^	↑ *^,1^ (*Cyfip1*)	ND	ND
**Neurite length**	↑/normal	↑	↓	↓	↓	↓	↑/↓ *^,1^ (*Cyfip1*)	ND	ND
**Spine density**	↑ *^,1^/↓ *^,1^/normal	↑ *^,1^	↓	↑/↓/normal	↓ *^,1^	↓	↑ * (*Cyfip1*)	↑	↑
**Spine length**	↑/↓ *^,1^/normal	↑	ND	↑/↓/normal	↓	ND	ND	ND	ND
**Immature spine morphology**	↓ *^,1^/↑	↑/↓	ND	↑ *^,3,4^	Abnormal *^,1^	↑	↓ (*Cyfip1*)	ND	ND
**LTP**	↓	↑/↓	↓	↑/↓	↓ *^,1,3^	↓	↓ (*patDp/+*)	↑ *^,7^	↓
**mGluR-LTD**	↓ *^,1^	↓	ND	↑ *^,3,4^	↑	ND	ND	↑ *^,7^	ND
**Protein synthesis**	↓ *^,1^/↑ *^,1^	↑	↑ *^,1^	↑ *^,1,2,3,4^	ND	↓ *^,5,6^	ND	↑ *^,7^	↑ *^,8^

↑ = increased; ↓ = decreased; * Phenotypes were rescued by: 1, mTORC1 inhibition (rapamycin); 2, phosphatidylinositide 3-kinase (PI3K) inhibition; 3, ribosomal protein S6 kinase 1 (S6K1) depletion; 4, eukaryotic translation initiation factor (eIF) 4E phosphorylation reduction; 5, insulin-like growth factor 1 (IGF-1) and/or brain-derived neurotrophic factor (BDNF); 6, phosphatase and tensin homolog (PTEN) depletion; 7, 4EGI-1, an inhibitor of eIF4E-eIF4G interaction; 8, p110δ inhibition; *cytoplasmic FMR1 interacting protein 1* (*Cyfip1)* = transgenic mice and/or cultured neuronal cells overexpressing *Cyfip1*; *patDp/+* = model mice for 15q11-13 duplication; ND = not determined; TSC = tuberous sclerosis complex; NF1 = neurofibromatosis type I; FXS = fragile X syndrome; AS = Angelman syndrome; RTT = Rett syndrome; LTP = long-term potentiation; mGluR-LTD = metabotropic glutamate receptor-mediated long-term depression.
